# Hospitalization, mortality and public healthcare expenditure in Brazil during the COVID-19 crisis: vulnerabilities in the spotlight

**DOI:** 10.1590/1516-3180.2021.0496.23072021

**Published:** 2021-12-15

**Authors:** Israel Júnior Borges do Nascimento, Ana Luíza Matos de Oliveira, Paulo Henrique Costa Diniz, Maria de Fatima Leite, Graziella Lage Oliveira

**Affiliations:** I Clinical Pathologist (ClinPath). Medical Research Specialist, School of Medicine and University Hospital, Universidade Federal de Minas Gerais (UFMG), Belo Horizonte (MG), Brazil; and Medical Research Specialist, School of Medicine, Medical College of Wisconsin, Milwaukee, Wisconsin, United States.; II BEcon, PhD. Visiting Professor, Faculdade Latino-Americana de Ciências Sociais (FLACSO), São Paulo (SP), Brazil.; III MD, PhD. Adjunct Professor, Department of Internal Medicine, School of Medicine and University Hospital, Universidade Federal de Minas Gerais (UFMG), Belo Horizonte (MG), Brazil.; IV PharD, PhD. Full Professor, Department of Physiology and Biophysics, Institute of Biological Sciences, Universidade Federal de Minas Gerais (UFMG), Belo Horizonte (MG), Brazil.; V BPsych, PhD. Adjunct Professor, Department of Social and Preventive Medicine, School of Medicine, Universidade Federal de Minas Gerais (UFMG), Belo Horizonte (MG), Brazil.

**Keywords:** SARS-CoV-2, Brazil, Public Health, Data Management, Health Expenditures, Inequality, Hospitalization, All-cause mortality, COVID-19 crisis

## Abstract

**BACKGROUND:**

Multiple opinion-based communications have highlighted the actions of the Brazilian government during the pandemic. Nevertheless, none have appraised public data to identify factors associated with worsening of the healthcare system.

**OBJECTIVE:**

To analyze and collate data from public health and treasury information systems in order to understand the escalating process of weakening of Brazilian healthcare and welfare since the beginning of the severe acute respiratory syndrome coronavirus 2 (SARS-CoV-2) pandemic.

**DESIGN AND SETTING:**

Secondary data study conducted using multiple public databases administered by the Brazilian federal government.

**METHODS:**

We processed information from multiple national databases and appraised health and economic-related data.

**RESULTS:**

Based on our analyses, there were substantial reductions in inpatient hospital admissions and in the numbers of patients seeking primary care services, along with a decrease in immunization coverage. Moreover, we observed a considerable decline in government transfers to hospital services (reduction of 82.0%) and a diminution of public outlays in several healthcare-related subfunctions (“hospital and outpatient care”, “primary care”, “prophylactic and therapeutic support” and “epidemiological surveillance”). We observed an increase in the overall mortality rate over the period analyzed, especially regarding all group-based diseases. Notably, there were remarkable differences among geographic, racial, gender and other parameters, thus revealing the impact of vulnerabilities on COVID-19 outcomes.

**CONCLUSION:**

This assessment of documentation of public expenditure and the shrinkage of investment in sensitive areas of the healthcare system in Brazil emphasized areas that still require collective attention in order to guarantee national welfare.

## INTRODUCTION

Ever since 2020 began, governments worldwide have been implementing community, economic and health-related policies to tackle the hurdles resulting from the COVID-19 pandemic.^1^^,^^2^ These have included financial support packages to encourage investment and maintenance of local business, expanded and strengthened public healthcare and reasonable financial relief programs to socioeconomically vulnerable individuals.^3^^,^^4^^,^^5^^,^^6^ However, going against the global trends of continuous health and socioeconomic support to families and individuals experiencing any coronavirus-related impact, recent Brazilian government decisions have negatively affected millions of lives, particularly in impoverished macroregions.^7^^,^^8^^,^^9^^,^^10^

Over the last months, several reports in the popular media and editorials have reported on the anti-science discourse and anti-life decisions taken by the Brazilian government leader.^11^^,^^12^^,^^13^^,^^14^^,^^15^ These decisions included cessation or reduction of the “*Emergency Benefit*”. This has raised poverty and has forcibly led vulnerable individuals to suspend their social distancing measures. Moreover, divisive rhetoric that has exacerbated economic inequality and the caregiving crisis in Brazil has been disseminated.^16^^,^^17^

Nevertheless, to the extent of our knowledge, no previous reports have combined financial and health-related data from multiple public datasets to analyze the potential effects of decision-makers’ measures on general health. Thus, this study outlines, evaluates and combines data from public health and treasury information systems in order to understand the escalating process of weakening of the Brazilian healthcare system and worsening of welfare since the onset of the pandemic.

## OBJECTIVE

To analyze and collate data from public health and treasury information systems in order to understand the escalating process of weakening of Brazilian healthcare and welfare since the beginning of the severe acute respiratory syndrome coronavirus 2 (SARS-CoV-2) pandemic.

## METHODS

We designed and conducted this study through secondary data that originated from the Information Technology Department of the Brazilian National Health System (DATASUS), the health information system for primary healthcare (SISAB) and the Brazilian public expenditure portal (a platform dedicated to making public all expenditures of the federal government).^18^^,^^19^^,^^20^ DATASUS is an online interface governed by the Secretariat for Strategic and Participative Management of the Ministry of Health.^18^ It contains multiple information about procedures performed at primary, secondary and tertiary healthcare facilities.^18^ Additionally, SISAB is a strategy created by the Department of Family Health that has the aim of expanding information management and process automation, thereby improving conditions and improving work processes.^19^

We extracted information about the numbers of hospital admission authorizations, total cost of admissions, mortality rate and amounts of the federal government transfers. The data were stratified according to geographic region, race, type of medical care (elective or emergency), level of complexity and chapter of the International Classification of Diseases, 10^th^ edition (ICD-10).

We defined four health indicators as relevant primary healthcare performance measurements, in order to monitor the healthcare actions and services offered to society within the primary care level: 1. Mean percentage of diabetic individuals for whom measurements of glycated hemoglobin (HbA1c) were requested; 2. Mean percentage of hypertensive individuals for whom blood pressure levels were measured each semester; 3. Mean proportion of pregnant females who attended at least six prenatal consultations, among which the first consultation occurred no later than the 20^th^ gestational week; and 4. Immunization coverage rate. We also examined public healthcare-related expenditure by assessing the overall amounts paid in specific subareas/subfunctions (hospital and outpatient assistance, primary care, therapeutic and prophylactic support, general administrative tasks, epidemiological surveillance and others). Descriptive analysis was performed, and the variance over the period analyzed was calculated. The data were stored and processed using Microsoft Excel (Microsoft Corporation, Redmond, Washington, United States).

All quantitative data from 2020 were compared with baseline data from 2019. All monetary-related variables were deflated in accordance with the Expanded Consumer Price Index (IPCA), based on the 2019 values.^21^ One United States (US) dollar was equivalent to 5.32 Brazilian reais on May 25, 2020.

## RESULTS

**Figure 1.**
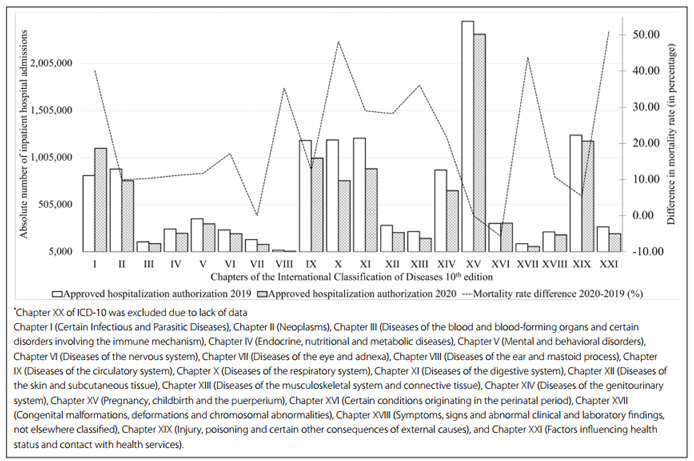
Absolute numbers of inpatient hospital admissions in Brazil, according to the chapters of the International Classification of Diseases 10^th^ edition (ICD-10)^*^, and the percentage difference in the mortality rate between the years 2019 and 2020.

Overall, there was a reduction in the number of inpatient hospital authorizations in 2020, compared with 2019 (mean decrease of 13.7%, ranging from 11.4% to 15.6%; Table 1 and Figure 1). The reduction was more pronounced in the northeastern and southern regions (-15.6% and -15.1%, respectively) and among white and indigenous individuals (-15.1% and -16.1%, respectively). Interestingly, black individuals were a racial group with a disproportionately large decrease in inpatient hospital authorizations (-3.4%), compared with other racial groups. Additionally, elective medical hospitalizations showed a substantial decline from 2019 to 2020 (-34.3%) with increases in the total cost of hospitalizations (+3.4%) and the all-cause mortality rate (+30.6%).

**Table 1.** Differences in hospitalization parameters, associated costs, all-cause mortality rates and federal complement for hospital services between 2019 and 2020, in the Brazilian National Health System

	Inpatient hospital admissions	Total cost (approved value of production)	Mortality rate	Federal complement for hospital services
	Δ % difference from 2019 to 2020			
Total	-13.7	3.4%	30.6	-81.9%
Geographic area				
North	-11.9	6.6%	44.6	-40.7%
Northeast	-15.6	2.2%	33.2	-85.3%
Southeast	-12.6	5.5%	29.0	-85.3%
South	-15.1	-1.1%	25.7	-85.0%
Center-West	-11.4	7.6%	32.8	-64.7%
According to race				
White	-15.1	0.8%	27.2	-83.9%
Black	-3.4	17.4%	35.5	-83.4%
“Pardo” (mixed)	-12.3	3.8%	30.3	-81.4%
Asian	-6.8	15.8%	36.2	-86.4%
Indigenous	-16.1	-0.8%	49.7	-81.9%
According to gender				
Male	-13.1	6.7%	32.5	-83.2%
Female	-14.2	0.07%	27.8	-81.0%
Type of medical care				
Elective	-34.3	-21.9%	83.6	-81.9%
Emergency and urgent	-8.0	12.7%	21.9	-
Level of complexity				
Medium	-13.5	10.7%	31.4	-80.7%
High	-16.6	-12.5%	14.3	-87.8%

Δ = difference between data from 2019 and 2020, in percentages. Data were obtained from the Department of Informatics of the Brazilian National Health System/Hospital Information System (*Departamento de Informática do Sistema Único de Saúde do Brasil/Sistema de Informações Hospitalares*, DATASUS-SIH).

Considering each chapter of the ICD-10, most groups showed a decrease in the number of inpatient hospital authorizations, except with regard to chapter I (infectious and parasitic diseases) and chapter XVI (disorders of the perinatal period). Nevertheless, as can be seen in Figure 1, the decrease in hospital admissions was not followed by any reduction in the specific-cause mortality rate (except with regard to chapters XVI, VII and XV). Additionally, there was an increase in deaths due to infectious and parasitic diseases (approximately 40.0%).

Regarding monetary variables, general hospitalization costs increased in most Brazilian macroregions, except in the southern region (-1.1%). In addition, hospitalization costs declined among individuals of indigenous origin (-0.8%), elective medical hospitalizations (-21.9%) and high-complexity care procedures (-12.5%). Regarding the general value of the federal complement of hospital services approved over the period analyzed, a substantial decrease in the amount transferred was noticed from 2019 to 2020 (-82.0%). Based on the public expenditure analysis (Table 2), the federal government spent nearly R$ 114 billion (9.5% of total federal expenditure) on healthcare in 2019. Of that, the subfunction “hospital and outpatient care” accounted for nearly R$ 57 billion, “primary care” about R$ 26 billion, “prophylactic and therapeutic support” about R$ 10 billion and “general administration” about R$ 7 billion. The full-year federal expenditure report for the fiscal year 2020 showed that there was an increase in the federal government’s direct outlays on healthcare (approximately R$ 150 billion, corresponding to 9.4% of total federal expenditure), particularly for the subfunction “general administration” (relative increase of 24.2%). Nevertheless, significant reductions in public expenditure in several healthcare-related subfunctions were noticed, including for “hospital and outpatient care” (relative reduction of 13.6%), “primary care” (relative reduction of 5.9%), “prophylactic and therapeutic support” (relative reduction of 2.0%) and “epidemiological surveillance” (relative reduction of 1.1%).

**Table 2.** Brazilian federal government public expenditure on health between 2019 and 2020

Subarea/subfunction	Expenditure in million reais (%)		Difference in million reais (relative % difference from 2019 to 2020)
	2019	2020	
Hospital and outpatient care	57,017.14 (49.9)	54,585.73 (36.2)	-2,431.41 (-4.2)
Primary care	26,404.33 (23.1)	25,813.42 (17.1)	-590.91 (-2.2)
Prophylactic and therapeutic support	10,968.56 (9.6)	11,365.96 (7.5)	397.40 (+3.6)
General administration	7,959.63 (6.9)	47,013.74 (31.2)	39,054.11 (+490.6)
Epidemiological surveillance	6,266.46 (5.4)	6,091.47 (4.0)	-174.99 (-2.7)
Others	5,565.96 (4.8)	5,592.95 (3.7)	26.99 (+0.04)
Total	114,182.11 (100)	150,463.29 (100)	36,281.18 (+31.7)

Difference refers to the row data from 2019 and 2020. The relative difference is expressed as a percentage and is calculated as follows: [difference from 2019 to 2020)/2019]. Data were obtained from DATASUS-SIH, SISAB and the Brazilian public expenditure portal. Values are expressed per R$ 1,000,000. US$ 1.00 = R$ 5.32 on May 25, 2021.

The all-cause mortality rate increased among all the major parameters analyzed (Figure 1). The greatest differences in all-cause mortality rate were observed in the northern region (+44.6%), among indigenous and male individuals (+49.7% and +32.5%, respectively), in elective medical procedures (+83.6%) and in medium-complexity care (+45.9%).

Among primary care performance variables, four main parameters were analyzed. Overall, the mean percentage of diabetic individuals for whom glycated hemoglobin (HbA1c) measurements were requested rose by 3.0% (5.3% in 2019 to 8.3% in 2020). Similarly, the mean percentage of hypertensive individuals for whom blood pressure levels were measured in each semester increased slightly from 2019 (3.0%) to 2020 (3.3%). Furthermore, an increase of 3.0% in the mean proportion of pregnant females who attended at least six prenatal consultations among which the first consultation occurred no later than the 20^th^ gestational week was observed over the period analyzed. However, immunization coverage dropped from 73.4% in 2019 to 66.6% in 2020.

## DISCUSSION

This study demonstrates that although there has been an overall increase in the transfer of funds to the healthcare system in Brazil since the onset of the pandemic, no immediate improvement in national health-related variables has been noticed. We observed contrasting reductions in the numbers of inpatient hospital authorizations in relation to different geographic, racial and gender-related features. Remarkably, there was an unequal decline in hospital admissions among black individuals. Immunization coverage was the only primary care performance benchmark that declined from 2019 to 2020. A significant reduction in hospital admissions was observed with regard to most chapters of the ICD-10, except for infectious and parasitic diseases and disorders of the perinatal period, but this was not strictly associated with the decrease in the specific-cause mortality rate. Considering the abovementioned parameters, recent political decisions may have impacted Brazil over the course of the coronavirus pandemic, with regard to national welfare.

Our results have implications for policymakers and medical professionals, given that most individuals in situations of poverty were significantly more affected by the pandemic. Our findings suggest that vulnerable populations (including indigenous and black individuals) and people living in socioeconomically vulnerable settings (such as in the Northeast and North of Brazil) may have been negatively overwhelmed by the pandemic, potentially because these populations have historically had inadequate access to care, generally have precarious work conditions, with informal employment that often cannot be accommodated to remote work. Similar results have been reported from several cohorts and worldwide cross-sectional studies.^22^^,^^23^^,^^24^^,^^25^ The study by Rocha et al. demonstrated the correlation between socioeconomic vulnerability and the disorderly spread of the disease and mortality in Brazil.^22^

Furthermore, according to Garg et al., among COVID-19 deaths registered in New York City for which race and ethnicity data were obtainable, mortality rates were considerably higher among black or African-American populations (92.3 deaths per 100,000 individuals) and among Hispanic or Latino individuals (45.2 deaths per 100,000 individuals).^25^ Likewise, based on data from the Mexican Ministry of Health, Ibarra-Nava et al. identified that the proportion of deaths caused by COVID-19 among indigenous communities was higher than in non-indigenous populations (16.5% versus 11.1%, respectively).^24^ Even though recent studies have emphasized the late impact of the pandemic among socioeconomically susceptible individuals, there is still a need for additional studies addressing the effects of the pandemic among groups, in order to prevent and limit the spread of coronavirus among people who are at greater risk of poorer outcomes.

The mean 13.7% reduction in inpatient hospital admissions was accompanied by a lower number of hospitalizations for elective medical procedures (mean reduction of 34.3%) and an increase in the all-cause mortality rate (30.6%). Similar findings have been reported in the literature, including among developed countries such as Italy, Spain, the United States and the United Kingdom.^26^^,^^27^^,^^28^^,^^29^ For instance, in the United States, cardiac catheterization due to laboratory ST-segment elevation myocardial infarction activation was reduced by 38.0%, and this was similar to results found in Spain (40.0% reduction).

Regarding oncology services, a recent systematic review showed that interruption of cancer treatment at any stage was reported by up to 77.5% of the patients enrolled at the services included in the surveys, and that this was usually associated with reduced service availability.^30^ In general, the reasons for this reduction in services may involve interruption of clinical and surgical procedures, including those with diagnostic purposes, and a representative decrease in transplantation activities. This ultimately implies an increasing accumulation of patients with chronic conditions but without care, which would be in addition to the historical repressed demand for elective surgeries. This situation may be exceedingly harmful, and is aggravated by the reduction of 81.9% in federal complementation of resources destined for hospitalizations. Thus, we reiterate and advocate that there is a need to maintain access to healthcare, particularly for patients with comorbidities, in order to decrease morbidity and mortality among this considerable proportion of the whole population.

We found that there had been considerable reductions in public expenditure in relation to important subfunctions of the Ministry of Health (hospital and outpatient care, primary care, prophylactic and therapeutic support and epidemiological surveillance). Together, the target for the strategy for reductions in federal government outlays totaled more than R$ 3.1 billion. Nevertheless, federal transfers within the “general administrative” subfunction have increased nearly fivefold, which represents an unprecedented rise since 2016. This specific governmental subfunction coordinates the management and maintenance of other government bodies, including federal employees and unit administration payments, and does not necessarily correlate with improvement of the overall healthcare system. Contrariwise, in nations with better-coordinated pandemic governance arrangements, including improvement of outpatient services, social supportive policies and primary care, the investment in areas specifically oriented towards healthcare have increased during the pandemic.^31^^,^^32^^,^^33^^,^^34^

We observed that there was a high excess mortality rate in undeveloped and socially vulnerable areas, and also among indigenous, Asian and black individuals. Our mortality results were similar to those reported for Asian, European, South and North American and African countries.^35^^,^^36^^,^^37^^,^^38^^,^^39^ For instance, in a study conducted in the United States, in which 3,135 counties from different datasets were enrolled, individuals living in “poor housing conditions” had a 50.0% higher risk of contracting SARS-CoV-2 and a 42.0% higher risk of dying because of the viral infection. Correspondingly, in Stockholm, Sweden, an excess mortality rate was noticed among people living in socioeconomically deprived areas (excess mortality rate ranging from 162% to 178%). Interestingly, our results showed that an increase in hospitalization costs is unlikely to translate into a reduced mortality rate. Therefore, governmental officials need to understand that the achievement of highly impactful healthcare outcomes is often not purely associated with the quantity of resources available but, rather, with how the system is reorganized.

The mean 6.8% reduction in the vaccination coverage was the only primary care indicator that demonstrated significant variation, over the period analyzed. However, the performance of all three additional parameters was classified as insufficient (< 20% of the target population had comorbidity-specific check-ups over the period). This result suggests that further actions are required in order to strengthen the primary care system and related activities, and to establish a more efficient healthcare policy, so as to prevent the development and worsening of most diseases.

## CONCLUSION

The scenario described here highlights how healthcare inequalities have become more pronounced during the COVID-19 pandemic due to underfunding of the healthcare system and social policies in general, as well as due to the dynamic of the pandemic, which has increased historical vulnerabilities. At times, the actions and, at times, inaction of the Brazilian government have gone against worldwide evidence about the importance of continuous primary healthcare systems, health surveillance and social protection programs. These are critical for improving equity and access, healthcare performance and health outcomes, and for identifying public health emergencies, particularly during crises like the ongoing pandemic.

## References

[1] Cheng C, Barceló J, Hartnett AS, Kubinec R, Messerschmidt L (2020). COVID-19 Government Response Event Dataset (CoronaNet v.1.0). Nat Hum Behav.

[2] Haldane V, De Foo C, Abdalla SM (2021). Health systems resilience in managing the COVID-19 pandemic: lessons from 28 countries. Nat Med.

[3] Ueno H, Bengali S (2021). A town in Japan spent Covid relief funds on a giant squid statue. The New York Times.

[4] Wallonie Lutter contre la pauvreté. La Wallonie répond à vos questions.

[5] U.S. Department of the Treasury Covid-19 Economic Relief.

[6] GOV.UK Financial support for businesses during coronavirus (COVID-19).

[7] Simões E, Paraguassu L (2021). Brazil business elite blast Bolsonaro, who remains unconvinced on COVID-19 restrictions. Business News at Reuters.

[8] Brum E (2020). Brazil’s message to the world: our president is wrong about coronavirus | Eliane Brum. The Guardian.

[9] Nassif Pires L, Barbosa de Carvalho L, Lederman Rawet E (2020). Multi-dimensional inequality and COVID-19 in Brazil. Investigación Económica.

[10] Barberia LG, Gómez EJ (2020). Political and institutional perils of Brazil’s COVID-19 crisis. Lancet.

[11] Dyer O (2020). Covid-19: Bolsonaro under fire as Brazil hides figures. BMJ.

[12] Taylor L (2021). Covid-19: Investigation probes Bolsonaro’s role in Brazil’s failed pandemic response. BMJ.

[13] Daniels JP (2021). Health experts slam Bolsonaro’s vaccine comments. Lancet.

[14] Escobar H (2021). Researchers face attacks from Bolsonaro regime. Science.

[15] Ponce D (2020). The impact of coronavirus in Brazil: politics and the pandemic. Nat Rev Nephrol.

[16] Nassif-Pires Luiza, Cardoso Luísa, Oliveira Ana Luíza Matos de Gênero e raça em evidência durante a pandemia no Brasil: o impacto do Auxílio Emergencial na pobreza e extrema pobreza. Nota de Política Econômica nº 010.

[17] Ahmed F, Ahmed N, Pissarides C, Stiglitz J (2020). Why inequality could spread COVID-19. Lancet Public Health.

[18] Ministério da Saúde. DATASUS - Departamento de Informática do SUS Informações de Saúde (TABNET).

[19] Ministério da Saúde. SISAB - Sistema de Informação em Saúde para a Atenção Básica Indicadores de Desempenho.

[20] Controladoria-Geral da União Despesa Pública - Portal da transparência (2019 and 2020).

[21] Instituto Brasileiro de Geografia e Estatística (IBGE) Índice Nacional de Preços ao Consumidor Amplo - IPCA.

[22] Rocha R, Atun R, Massuda A (2021). Effect of socioeconomic inequalities and vulnerabilities on health-system preparedness and response to COVID-19 in Brazil: a comprehensive analysis. Lancet Glob Health.

[23] Charlier P, Varison L (2020). Is COVID-19 being used as a weapon against Indigenous Peoples in Brazil?. Lancet.

[24] Ibarra-Nava I, Flores-Rodriguez KG, Ruiz-Herrera V (2021). Ethnic disparities in COVID-19 mortality in Mexico: A cross-sectional study based on national data. PLoS One.

[25] Garg S, Kim L, Whitaker M (2020). Hospitalization Rates and Characteristics of Patients Hospitalized with Laboratory-Confirmed Coronavirus Disease 2019 - COVID-NET, 14 States, March 1-30, 2020. MMWR Morb Mortal Wkly Rep.

[26] Mauro V, Lorenzo M, Paolo C, Sergio H (2020). Treat all COVID 19-positive patients, but do not forget those negative with chronic diseases. Intern Emerg Med.

[27] Garcia S, Albaghdadi MS, Meraj PM (2020). Reduction in ST-Segment Elevation Cardiac Catheterization Laboratory Activations in the United States During COVID-19 Pandemic. J Am Coll Cardiol.

[28] Al-Quteimat OM, Amer AM (2020). The Impact of the COVID-19 Pandemic on Cancer Patients. Am J Clin Oncol.

[29] Rodríguez-Leor O, Cid-Álvarez B, Ojeda S (2020). Impacto de la pandemia de COVID-19 sobre la actividad asistencial en cardiología intervencionista en España. REC Interv Cardiol.

[30] Riera R, Bagattini ÂM, Pacheco RL (2021). Delays and Disruptions in Cancer Health Care Due to COVID-19 Pandemic: Systematic Review. JCO Glob Oncol.

[31] Australian Government Department of Health (2020). Government response to the COVID-19 outbreak. Australian Government Department of Health.

[32] Australian Government Department of Health Strengthening Primary Care - COVID-19 primary health response.

[33] Krist AH, DeVoe JE, Cheng A, Ehrlich T, Jones SM (2020). Redesigning Primary Care to Address the COVID-19 Pandemic in the Midst of the Pandemic. Ann Fam Med.

[34] Park S, Elliott J, Berlin A, Hamer-Hunt J, Haines A (2020). Strengthening the UK primary care response to covid-19. BMJ.

[35] Calderón-Larrañaga A, Vetrano DL, Rizzuto D (2020). High excess mortality in areas with young and socially vulnerable populations during the COVID-19 outbreak in Stockholm Region, Sweden. BMJ Glob Health.

[36] Zhou F, Yu T, Du R (2020). Clinical course and risk factors for mortality of adult inpatients with COVID-19 in Wuhan, China: a retrospective cohort study. Lancet.

[37] Ahmad K, Erqou S, Shah N (2020). Association of poor housing conditions with COVID-19 incidence and mortality across US counties. PLoS One.

[38] Mena GE, Martinez PP, Mahmud AS (2021). Socioeconomic status determines COVID-19 incidence and related mortality in Santiago, Chile. Science.

[39] Wallace LJ, Nouvet E, Bortolussi R (2020). COVID-19 in sub-Saharan Africa: impacts on vulnerable populations and sustaining home-grown solutions. Can J Public Health.

